# Potential Compounds for Oral Cancer Treatment: Resveratrol, Nimbolide, Lovastatin, Bortezomib, Vorinostat, Berberine, Pterostilbene, Deguelin, Andrographolide, and Colchicine

**DOI:** 10.1371/journal.pone.0141719

**Published:** 2015-11-04

**Authors:** Saurabh Bundela, Anjana Sharma, Prakash S. Bisen

**Affiliations:** 1 Defence Research Development Establishment, Defence Research Development Organization, Ministry of Defence, Govt. of India, Gwalior, Madhya Pradesh, India; 2 Department of Postgraduate Studies & Research in Biological Sciences, Rani Durgavati University, Jabalpur, Madhya Pradesh, India; 3 School of Studies in Biotechnology, Jiwaji University, Gwalior, Madhya Pradesh, India; Columbia University Medical Center, UNITED STATES

## Abstract

Oral cancer is one of the main causes of cancer-related deaths in South-Asian countries. There are very limited treatment options available for oral cancer. Research endeavors focused on discovery and development of novel therapies for oral cancer, is necessary to control the ever rising oral cancer related mortalities. We mined the large pool of compounds from the publicly available compound databases, to identify potential therapeutic compounds for oral cancer. Over 84 million compounds were screened for the possible anti-cancer activity by custom build SVM classifier. The molecular targets of the predicted anti-cancer compounds were mined from reliable sources like experimental bioassays studies associated with the compound, and from protein-compound interaction databases. Therapeutic compounds from DrugBank, and a list of natural anti-cancer compounds derived from literature mining of published studies, were used for building partial least squares regression model. The regression model thus built, was used for the estimation of oral cancer specific weights based on the molecular targets. These weights were used to compute scores for screening the predicted anti-cancer compounds for their potential to treat oral cancer. The list of potential compounds was annotated with corresponding physicochemical properties, cancer specific bioactivity evidences, and literature evidences. In all, 288 compounds with the potential to treat oral cancer were identified in the current study. The majority of the compounds in this list are natural products, which are well-tolerated and have minimal side-effects compared to the synthetic counterparts. Some of the potential therapeutic compounds identified in the current study are resveratrol, nimbolide, lovastatin, bortezomib, vorinostat, berberine, pterostilbene, deguelin, andrographolide, and colchicine.

## Introduction

Despite of great progress made in the field of medical science, there are still, over 32.6 million people living with cancer worldwide. There were 8.2 million cancer deaths in 2012 worldwide, out of which, 0.68 million people died from cancer in India [1]. Cancer, which was once thought to be a disease prevalent in developed nations, has now spread across the world, in fact, recent cancer statistics shows that 65% (5.3 million) of all cancer-related deaths were reported from less developed countries [1]. This is definitely a dismal development in countries which are ill-equipped to fight complex disease like cancer. The prevalence and/or incidence rate of cancer-types vary significantly between different countries, for example, oral cancer, which is less common in developed countries, is ranked in the top three causes of cancer-related deaths among men in South Asian countries like India, Bangladesh, and Sri Lanka. The heterogeneity in the distribution of the prevalence of cancer-types between developed and less developed countries implies that the progress made in the area of cancer treatment, by developed countries cannot be efficiently applied in less developed countries. There is a wide range of treatment options available for lung, prostate and breast cancer, that are more prevalent in developed countries, however, treatment options are very limited, for cancers like oral cancer, which is relatively rare in developed countries. Factors like the high usage of tobacco in various forms, inability to diagnose cancer in early stage, and limited treatment options, are responsible for the high mortality rate associated with oral cancer. Oral cancer is currently managed through surgery, radiation therapy and chemotherapy [2].

**Fig 1:**
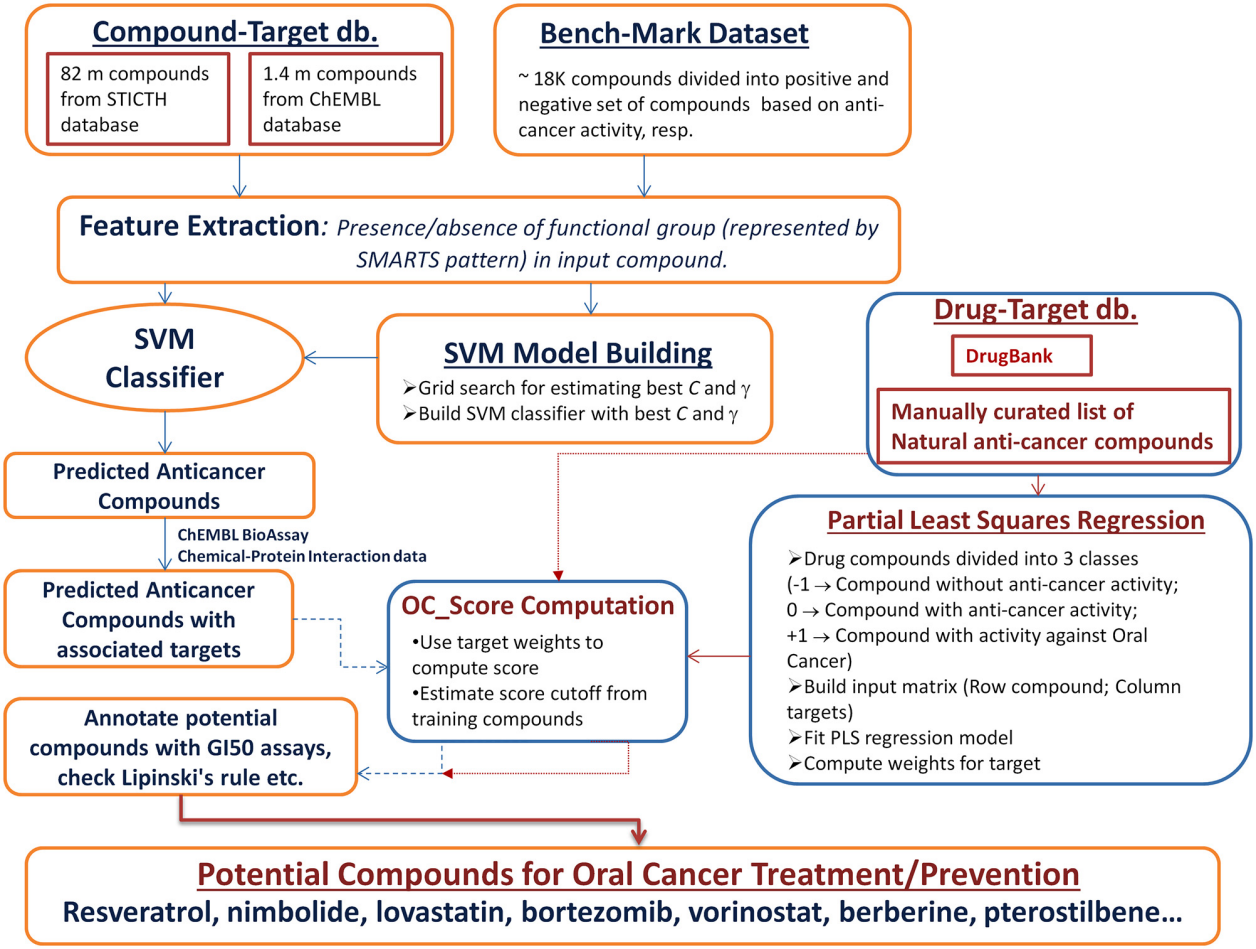
Process flow of identification of potential compounds for oral cancer treatment.

The current study, attempts to identify potential anti-cancer compounds for treatment of oral cancer. The availability of millions of bioactive compounds in publicly available databases like NCBI-PubChem and ChEMBL, offers great opportunity to mine the pool of compounds, based on attributes desired in the therapeutic area of interest. We have interrogated over 84 million compounds from databases like NCBI-PubChem, ChEMBL for the potential activity against oral cancer. A custom support vector machine (SVM) classifier was built for the prediction of anti-cancer activity among a pool of compounds. Features used for training and testing the SVM classifier, were derived from functional groups present in the compounds, which were used in model building and prediction process, respectively. The protein bioassay records for a compound were used to associate targets for anticancer compound predicted by SVM classifier. The target profile of the therapeutic compounds from the DrugBank database, and manually curated list of natural anti-cancer compounds, were used for building regression model, which was subsequently used for the computation of scores specific to oral cancer. The list of potential compounds was annotated with corresponding physicochemical properties, cancer specific bioactivity evidences, and literature evidences. Different analytical methods have been integrated to enable logical selection of the potential therapeutic compounds for oral cancer (Fig 1).

The current study presents a logical framework to find potential compounds for treatment of oral cancer, based on large-scale mining of reliable compound- and bioactivity- databases. The structural and target level patterns, shared by the compounds targeting the common pathology, were used in the current study for selection of the potential compounds for oral cancer.

## Materials and Methods

### Data Sources

#### Drug-Target data

DrugBank (version 4.0) [3] was used as a reference database to collect comprehensive information about drug-drug target information. The ‘drugbank.xml’ file was downloaded from download section of DrugBank (http://www.drugbank.ca/downloads); it was parsed by custom perl scripts to extract drug, along with its associated information like indication area, targets, SMILES string [4]. The indication area(s) associated with a drug is represented as free text in DrugBank, which poses algorithmic challenge for the process of automated association of drug with its indication area(s). In the current study, we have mapped diseases or indication area associated with drug to its corresponding ICD10 disease code [5], [6] (http://apps.who.int/classifications/icd10/browse/2010/en can be referred for detailed mapping between ICD10 disease code to related diseases).

The file ‘drug-disease_TTD2013.txt’, available from the download section of Therapeutic Target Database (TTD) [7], had been used for drug-disease mapping. This file can be used for unambiguous association of drug with its indication area(s). The files, ‘drug_links.csv’ and ‘TTD_crossmatching.txt’ (TTD), were used to retrieve mapping between DrugBank ID to TTD Drug ID. The data for the approved drugs along with its associated information, like drug target, ICD10 disease classification and SMILES string, was extracted from ‘drugbank.xml’ file. The data of the drugs was segregated into two group, anticancer drugs and other drugs, which is available as online supplementary material–‘DB_cancer.txt’ (see S1 Text) and ‘DB_others.txt’ (see S2 Text), respectively. DrugBank represents target information as UniProt ID, which was mapped into its corresponding Entrez Gene ID and Gene Symbol (based on mapping provided in ‘HUMAN_9606_idmapping_selected.tab’ and ‘gene_info’ files which can downloaded from ftp sites ftp://ftp.uniprot.org/pub/databases/uniprot/current_release/knowledgebase/idmapping/by_organism/HUMAN_9606_idmapping_selected.tab.gz, and ftp://ftp.ncbi.nlm.nih.gov/gene/DATA/gene_info.gz, respectively).

In the current study, ICD10 disease codes ‘C00 to C06’ were considered to represent oral cancer. While reviewing the information of anticancer drugs, we noticed that there are many drugs which are mapped against ICD10 disease code ‘C00-C96’, which is a non-specific disease code for malignant neoplasms. We could not find any drug in DrugBank database, which was indicated to treat oral cancer; therefore, we extended our search to the literature database (NCBI PubMed), and found evidence to control the growth of oral cancer cells by couple of drugs like erlotinib [8], [9], vandetanib [10] and gefitinib [8], [11]. The ICD10 disease code mapped for these drugs were updated manually, to include ‘C00-C06’ as drug indication in ‘DB_cancer.txt’ (see S1 Text). We realized that such a low representation of drugs for oral cancer treatment in public databases, would act as a bottleneck in downstream predictive data mining processes; this prompted us to extend our search beyond compound databases like DrugBank.

Nature is a gold-mine for treatment of various diseases, including cancer, which is evident from the fact that the majority of existing anticancer drugs are either natural products or their chemical derivatives [12]-[14]. We compiled the list of plant based anti-cancer natural compounds by manually mining literature databases like PubMed, and also used Google Scholar to search articles, not indexed with PubMed. A total of 269 articles were referred to collect the data about plant based natural compounds, active against over 25 different cancer types. We collected data for 377 compounds from these articles. The list of plant based compounds with anti-cancer activity was further annotated with associated attributes like PubChem Compound ID (cid), SMILES string, molecular targets. Target information was not present for all the compounds in the base set of articles (269 articles), therefore we further referred 315 more articles to collect target information of un-annotated compounds. The list of plant based natural anti-cancer compounds complied in the current study consists of 30 compounds with growth inhibitory activities against oral cancer cells. The list of plant based natural compounds active against various cancers obtained in the current study, can be found as online supplementary material–‘Natural_Anticancer_list.txt’ (see S3 Text), which contains links to research articles that were used to infer anti-cancer activities of compounds against particular cancer-type, and it also contains reference to articles which were used to infer compound to target association. This is a manually curated list, which can be of great use to researchers working in the field of plant based natural anti-cancer compounds. The data of ‘Natural_Anticancer_list.txt’ (see S3 Text) was further rearranged in a format similar to files obtained after mining DrugBank (see S1 and S2 Texts) to make it amenable for downstream data mining processes; this file can be found as online supplementary material–‘Nat_Anticancer.txt’ (see S4 Text).

#### Compound-Target Data sources

*ChEMBL—Compound Database*. ChEMBL is a freely available database of drug-like bioactive compounds [15]. The compound information present in this database is linked with bioactivity measurements, which are manually extracted from primary published literature. In the current study, we have utilized compound repository of ChEMBL (version 19.0) to be used for prediction of anti-cancer activity. We downloaded MySQL dump of ChEMBL and created a local database (ftp://ftp.ebi.ac.uk/pub/databases/chembl/ChEMBLdb/latest/chembl_19_mysql.tar.gz).

In the current study, we used perl libraries DBI and DBH for interfacing with ChEMBL database, created in locally installed MySQL. Perl scripts were written to extract data from the ChEMBL database. We extracted SMILES string along with ChEMBL id from the database with the help of following SQL query—“*select c*.*canonical_smiles*, *m*.*chembl_id from compound_structures c*, *molecule_dictionary m where c*.*molregno = m*.*molregno*”. A total of 1404752 compounds (i.e. ~1.4 million compounds) along with their SMILES strings were extracted from the database.

*STITCH—Chemical-Protein Interaction Database*. STITCH is a chemical-protein interaction database which integrates information about interactions from metabolic pathways, crystal structures, binding experiments and drug—target relationships [16]. In the current study, we have downloaded latest dataset from the STITCH database (version 4.0). Following files were downloaded from the download section of STITCH:

1. http://stitch.embl.de/download/protein_chemical.links.v4.0/9606.protein_chemical.links.v4.0.tsv.gz → Chemical-Protein Interaction data which contain over 4.5 million records. Chemicals are derived from the PubChem compound database, and proteins are represented by Ensembl protein identifiers.
2. http://stitch.embl.de/download/chemicals.v4.0.tsv.gz → Contains STITCH compound’s chemical structure information in the form of SMILES string. It contains 82841024 (i.e. ~ 82.84 million) compound records.
3. ftp://ftp.ncbi.nlm.nih.gov/gene/DATA/gene2ensembl.gz → Contains mapping between Ensembl protein identifier to NCBI-Entrez Gene ID.

### Anticancer Activity Prediction

Compound dataset collected from ChEMBL (1.4 million compounds) and STITCH (82.8 million compounds) was checked for the possible anti-cancer activity. It is to be noted that each compound record in STITCH database does not correspond to a unique molecule, i.e. there could be more than one record representing different stereo-isomers for a single compound [16]. In the current study, we have considered each record as a separate compound for prediction of anti-cancer activity, and duplicate compounds were removed from the list of compounds predicted to be active anti-cancer compounds. This was done to optimize the memory requirement for the task of identifying duplicates in a large pool of compounds. In the current study, we have used two methods for prediction of anti-cancer activity of almost 84 million compounds, (i) CDRUG [17] and (ii) a custom build support vector machine (SVM) classifier.

#### Benchmark Dataset

Benchmark dataset prepared for prediction of anti-cancer activity by Li et al. [17] was used in the current study. This dataset is from the NCI-60 Developmental Therapeutics Program (DTP) project [18]. The details of protocol used to create the benchmark dataset, can be found in primary published article [17]. The dataset consist of more than 18,000 compounds, divided into active and inactive anticancer compounds. The benchmark dataset can be downloaded from http://bsb.kiz.ac.cn/site_media/download/CDRUG/Benchmark.rar.

#### CDRUG

CDRUG is an analytical method for prediction of anticancer activity of chemical compound [17]. In the current study, we have downloaded and used the latest standalone version of CDRUG for anticancer activity prediction. This tool takes a list of SMILES string of query compounds as an input and generates ranked list consisting of various scores and p value. In the current study, we have considered the cutoff p value of ≤ 0.05, as criteria to select compounds with anticancer activity. The algorithmic details of CDRUG can be found in primary publication [17].

#### Support Vector Machine (SVM) Classifier

In the current study, we have built SVM based model for the prediction of anticancer activity of chemical compound. Support Vector Machines are a useful tool for data classification, which has found its application in wide range of domains including computational biology. We have used software LIBSVM (version 3.18) [19] in our current study for SVM based classification. The SVM based classification task starts with the process of “model building”, in which data is divided into training and testing sets. Each instance in the training set contains one “target value” or “class label” (in our case it is either 1 or 0; where ‘1’ represents compound has anti-cancer activity and ‘0’, otherwise), and several “attributes” or “features”. The goal of SVM [20], [21] is to rigorously build a model (based on instances from training data) which predicts the target values / class labels of the instances from test data, given only attributes in the test data. In the current study, we selected ‘C-SVM’ (Multi-class classification) as SVM type, and radial basis function (RBF) as a kernel type for building anti-cancer activity prediction model. RBF kernel was chosen on the basis of its popularity, robustness, and the fact that other kernels available with LIBSVM are special cases of RBF under certain parameter [22], [23].

The process of classification with SVM involves following steps:

1. Model building: In the current study, we have used benchmark dataset [17] (see the section Benchmark Dataset) for building SVM prediction model. The rationale behind the selection of dataset common to that, used by CDRUG [17], was to compare prediction outcomes of two methods (CDRUG and SVM classifier) build from the same underlying dataset. The process of building model involves following sub-steps:
  - Feature extraction of training compounds and transformation of feature vector into SVM input format.
  - Cross validation based parameter estimation and building model with best parameters.
2. Prediction of query compounds:
  - Data processing of query compound(s).
  - Prediction of anti-cancer activity of query compound(s).

*Feature Extraction*. In the current study, the features were derived from the entities in the compound, which are responsible for defining its reaction mechanism, and are the contributing factor towards its activity. These entities can be of organic (i.e. ‘functional groups’) or inorganic (i.e. ‘metal ions’) in nature. Functional groups present in organic molecules had been used in the past to predict drug-target interaction networks [24], wherein authors had used 28 functional groups to characterize drugs. In addition to the functional group, metals also play a very important role in determining the activity of drugs, especially in the field of cancer drug, such as cisplatin, which can be regarded as a pioneer in the field of metal based anti-cancer drug [25]. The functional groups and metals present in a compound can be visualized as building block or substructure of a compound. SMARTS is a very powerful language for describing such molecular substructures [26]. SMARTS strings are typically used for substructure searching, to identify molecules based on pattern matching, either a singular string or as a group of SMARTS strings. In the current study, we rigorously prepared SMARTS strings of over 300 functional groups (including common metallic forms found in various drugs). We have followed the guidelines given by Daylight [26], while preparing these SMARTS strings.

**Fig 2:**
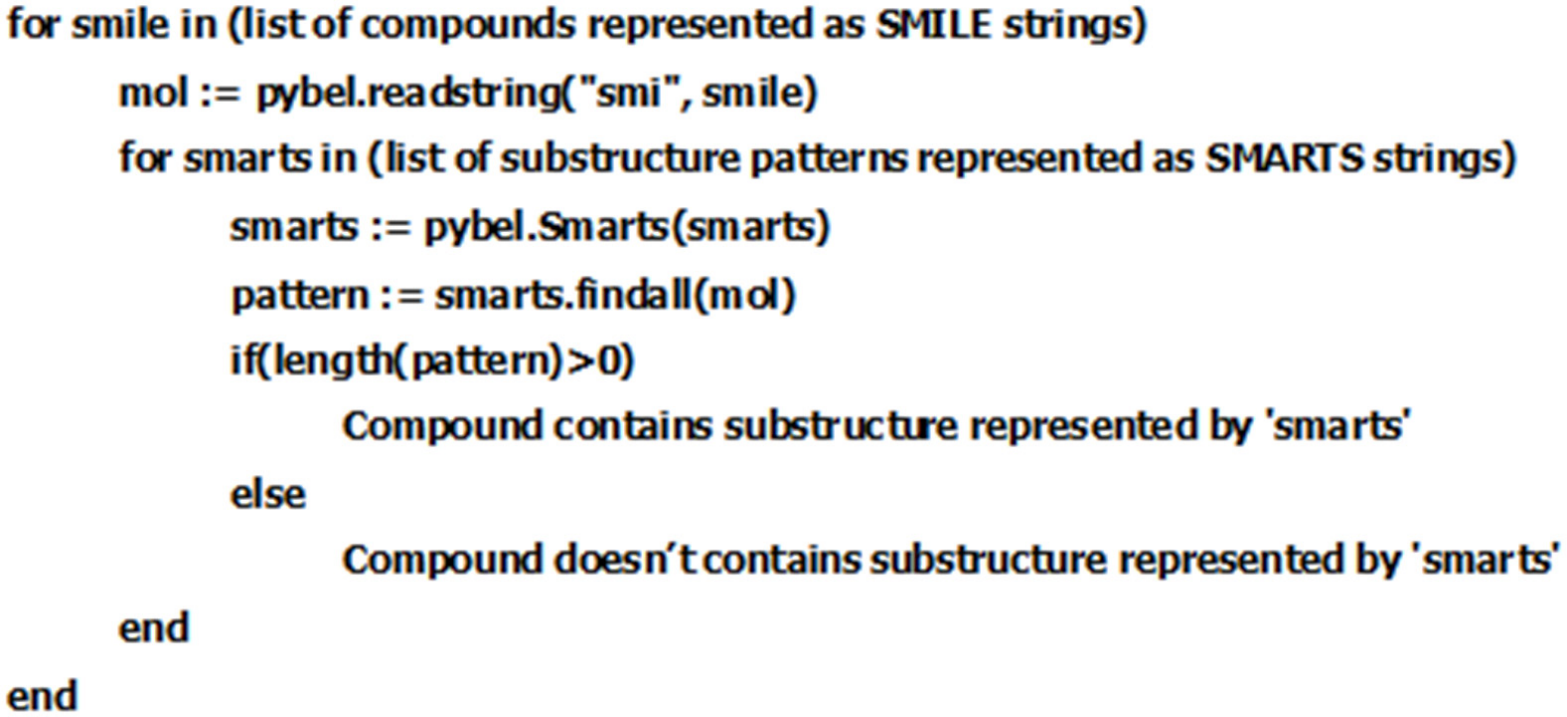
Pseudocode for substructure matching process.

Features were extracted from the training compounds, from the Benchmark dataset [17]. The dataset consist of over 18,000 compounds (positive- and negative-set) in SMILES format (refer to: http://bsb.kiz.ac.cn/site_media/download/CDRUG/Benchmark.rar). In the current study, we have used open-source python library Pybel [27] for finding substructures encoded as a SMARTS string in a query compound. Python script was written to automate the task of matching the list of SMARTS stings against the benchmark dataset (Fig 2).

On reviewing the extracted features of all compounds (positive and negative dataset), we observed that many of the substructures from our initial list of SMARTS string were not present in either of the dataset (i.e. positive- or negative-set), and therefore, they were excluded from the further downstream analysis process. The final list of SMARTS strings along with corresponding representative substructure (functional groups or metal ion) consisted of 228 SMARTS strings, which can be found as online supplementary material–‘SMARTS_pattern.txt’ (see S5 Text). At the end of this exercise, we obtained feature matrix of dimension M Γ N matrix; where ‘M’ corresponds to the number of compounds in benchmark dataset and ‘N’ corresponds to number of features/substructures (i.e. 228) used to prepare feature vector of a compound. This feature vector was transformed into a SVM format as given below:

<label> <index1>:<value1> <index2>:<value2>…

.

.

.

Where, each line contains an instance and is ended by a '\n' character. The <label> is an integer indicating the class label (1→Compound with anti-cancer activity and 0→Compound without anti-cancer activity). The pair <index>:<value> gives a feature (attribute) value: <index> is an integer starting from 1 and <value> is a real number (In the current study, <value> can be [0,1], where 0→indicates feature is absent in the compound, and 1→indicates feature is present in the compound). Indices must be in ascending order [19].

*Parameter Estimation and Model Building*. The RBF kernel has two parameters *C* and γ; for a given prediction problem, the value of these parameters is not known beforehand, and therefore, some kind of parameter search has to be done to estimate values of these parameters. The main objective of parameter search is to find good (*C*, γ), so that the prediction model will accurately predict activity of unknown compounds. Generally poorly optimized models tend to suffer with an overfitting problem, which refers to the condition when prediction model / classifier shows high accuracy with training data, but its accuracy drops drastically when used to predict unknown test data. Cross-validation is a technique which is applied to overcome the overfitting problem. In *n*-fold cross-validation, training dataset is divided into *n* subsets of equal size. Sequentially one subset is tested using the model, trained on the remaining *n*-1 subsets. In this way, each instance of the whole training set is predicted once, so that, the cross-validation accuracy is the percentage of data which are correctly classified.

**Fig 3:**
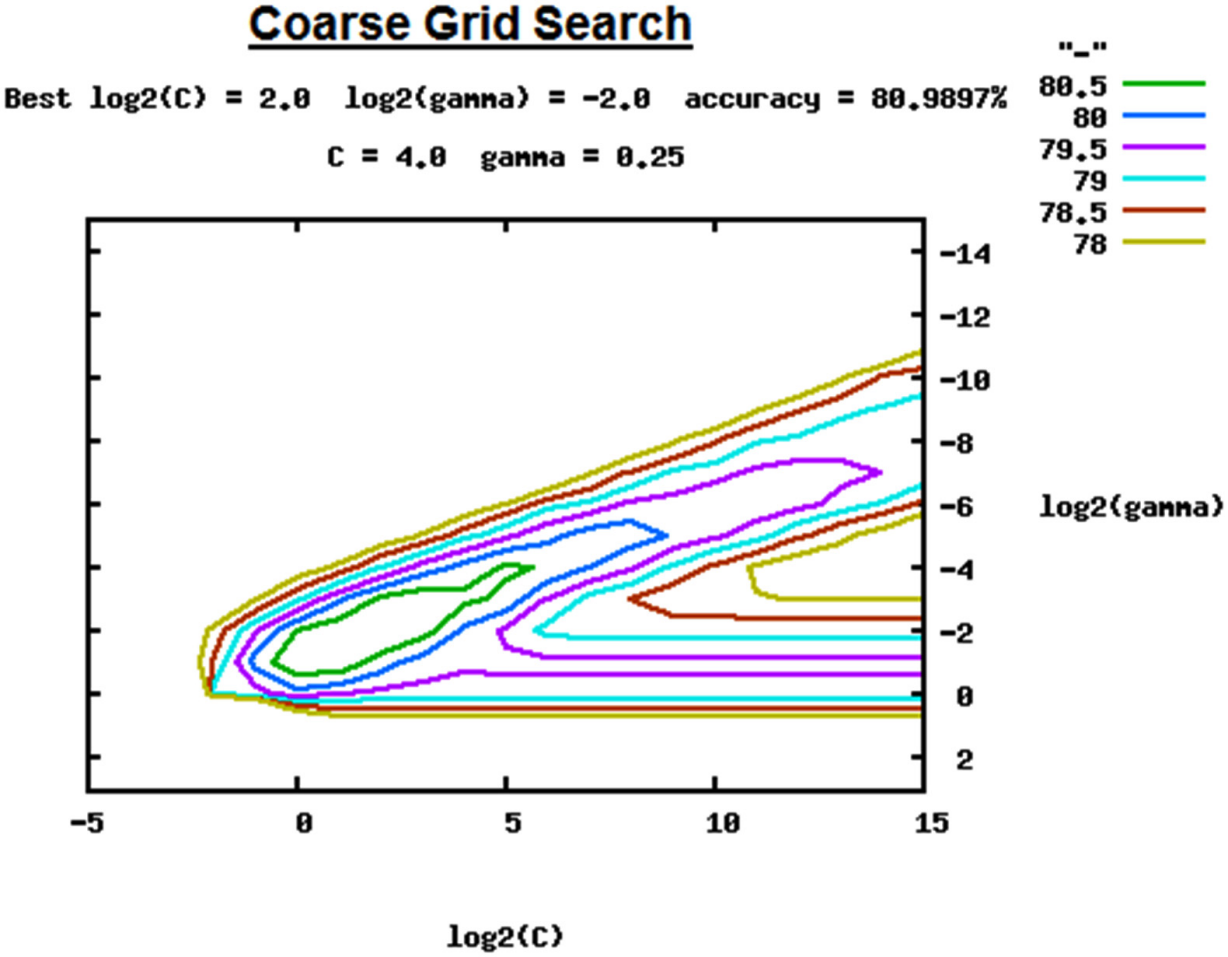
Coarse Grid Search for *C* and γ for parameter estimation.

In the current study, we performed an exhaustive grid—search on *C* and γ using 5-fold cross-validation. After feature extraction and data transformation of the benchmark dataset (see section Feature Extraction), we first did a coarse grid search for finding best *C* and γ using 5-fold cross-validation. We first started with coarse grid search with an exponentially growing sequence of *C* and γ (*C* = 2^−5^, 2^−4^, 2^−3^…, 2^14^, 2^15^ and γ = 2^−15^, 2^−14^….2^4^, 2^3^), which gave us best parameters (*C* = 2^2^ and γ = 2^−2^) with cross-validation accuracy of 80.99% (Fig 3). The parameters with cross-validation accuracy of over 80.5% are distinctly marked with green color in grid space of Fig 3, we next focused on fine grid search in this region.

**Fig 4:**
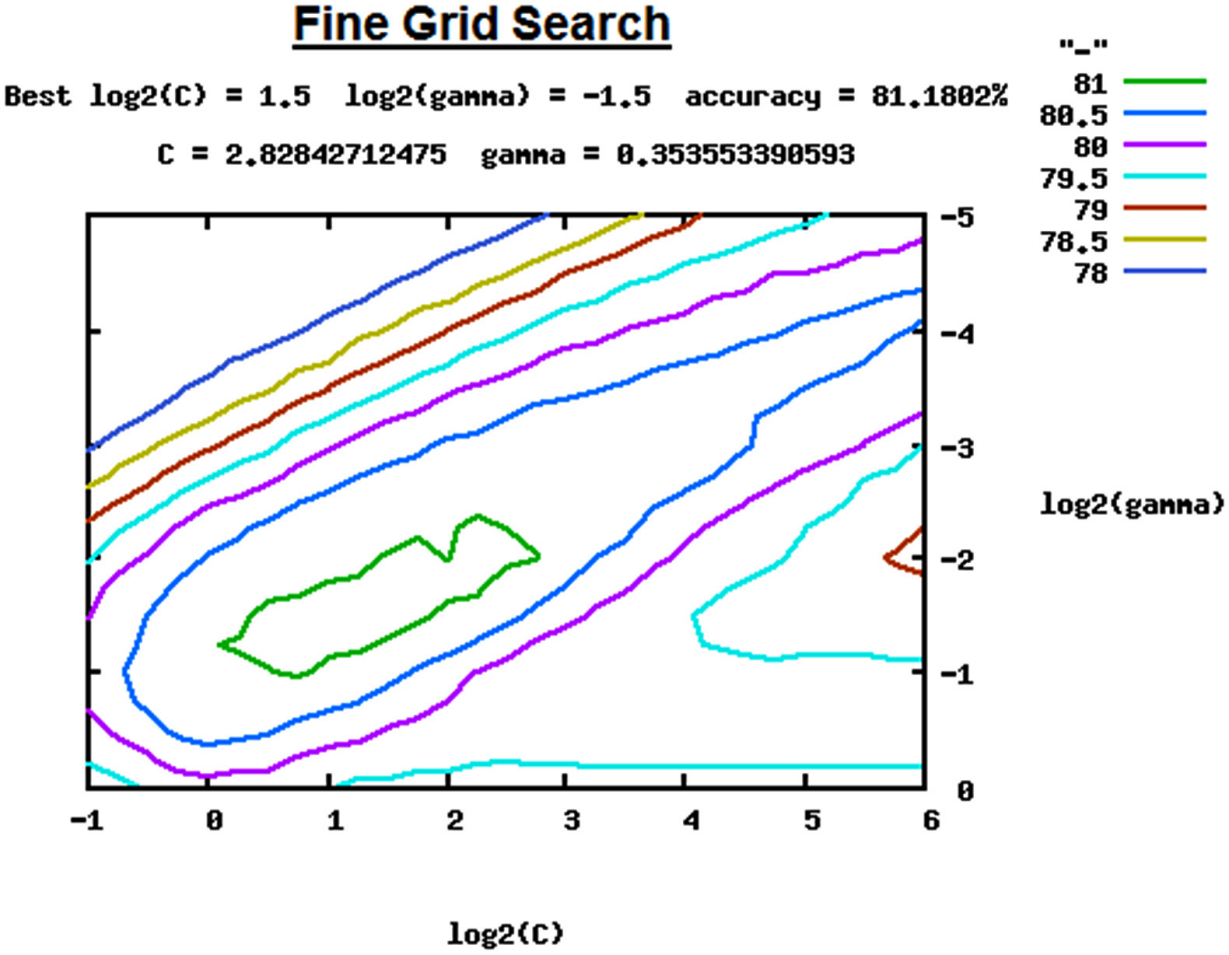
Fine Grid Search for *C* and γ for parameter estimation.

The fine grid search was conducted with a growing sequence of *C* and γ (*C* = 2^−1^, 2^−0.75^, 2^−50^…2^5.50^, 2^5.75^, 2^6^ and γ = 2^0^, 2^−0.75^….2^−4.50,^ 2^−4.75^, 2^−5^), which gave us best parameters (*C* = 2^1.5^ and γ = 2^−1.5^) with cross-validation accuracy of 81.18% (Fig 4). Whole training set (i.e. the transformed benchmark dataset with feature vectors) was used for building a final classifier with the best parameters (*C* = 2^1.5^ and γ = 2^−1.5^). The intermediate files generated during grid search, along with final classifier ‘*cancer*.*model*’ can be found as online supplementary material ‘Model_Build.zip’ (S6 Text). In the current study, the classifier ‘*cancer*.*model*’ was used in the subsequent SVM based prediction of anticancer activity. The exhaustive grid based parameter search was done with the help of the python script ‘grid.py’ available with LIBSVM package [19]. Computationally grid search is memory and CPU intensive task, in a parallel mode, it took almost 10 days to complete this task in 4 GB Intel^®^ Core^™^ i5 desktop installed with Linux operating system.

*Prediction Process*. The prediction of anticancer activity with SVM classifier ‘*cancer*.*model*’ for query compounds involves following steps:

1. Read list of ‘n’ number of query compounds.
2. Set initial index i = 1.
3. Preparation of feature vector for i^th^ query compound (as explained in section Feature Extraction). The feature vector D_i_[x1, x2….x228] for a i^th^ query compound, would be a binary vector representing the presence or absence of functional group/substructure in a query compound.
4. Check if ‘i’ is less than ‘n’, If yes then i = i+1 and go to step 3, else go to step 5.
5. Transform feature matrix into SVM input format and save as file “svm_input.dat”.
6. Predict with the following command:
  - ./svm-predict svm_input.dat cancer.model <output_name>

### Validation of Prediction Models

*Validation Dataset*. The accuracy of the methods for the prediction of anticancer activity (i.e. CDRUG and aforementioned SVM classifier) was tested with the help of the compound dataset, associated with their indication area without any ambiguity. The validation dataset used in the current study was randomly selected from the collection of DrugBank and natural plant based anti-cancer compounds (see section *Drug-Target data* for details about the primary dataset). We created a balanced dataset, which consisted of 526 compounds with anticancer activity (positive dataset), and 526 compounds without anticancer activity (negative dataset). The validation dataset, can be found as online supplementary material–‘cancer_nat_db_smi.txt’ (compounds with anti-cancer activity) (see S7 Text), and ‘others_smi.txt’ (compounds without anti-cancer activity) (see S8 Text).

The standalone version of CDRUG [17] was used to predict activity of validation dataset, the prediction results of CDRUG can be found in the file–‘validation_set_tab.txt’ (see S9 Text), which is available as online supplementary material. The svm classifier ‘cancer.model’ build in the current study, was also used to predict activity of validation dataset. The SVM based classification of validation dataset was achieved in following broad steps:

1. SMARTS string based computation of feature vector (see section Feature Extraction for detailed procedure). The result of the feature extraction process on validation dataset is available in file ‘Validation_dataset_features.txt’ (see S10 Text) as online supplementary material.
2. Transformation of feature vector into svm input format. The transformed feature matrix is available in file ‘Validation_dataset_dat.txt’ (see S11 Text) as online supplementary material.
3. SVM based prediction: Anticancer activity of validation dataset was predicted with following command of libsvm [19]: ./svm-predict Validation_dataset_dat.txt cancer.model Validation_dataset_out.txt.

The svm prediction result can be found in ‘Validation_dataset_out.txt’ (see S12 Text) as online supplementary material.

The prediction results obtained from CDRUG and SVM classifier were compared, based on following statistics:

$$Sensitivity=\frac{TP}{\left(TP+FN\right)}$$ (i)

$$Specificity=\frac{TN}{\left(TN+FP\right)}$$ (ii)

$$Accuracy=\frac{\left(TP+TN\right)}{\left(TP+FP+TN+FN\right)}\;\;$$ (iii)

where,

‘TP’ is True Positive.

‘TN’ is True Negative.

‘FP’ is False Positive.

‘FN’ is False Negative.

The sensitivity, specificity and accuracy statistics were calculated for the results obtained from both methods (Table 1). It can be seen that the overall accuracy of CDRUG method is slightly better than custom build SVM classifier, which can be mainly attributed to its exceptionally high specificity (~ 91%). The performance statistics of custom build SVM classifier can be regarded as balanced in terms of sensitivity (~ 61%) and specificity (~ 62%), whereas, the sensitivity of CDRUG observed as quite low (~40%) (Table 1).

**Table 1:** Comparison of Prediction Results.

Statistics	CDRUG	SVM
**TP**	214	323
**FN**	312	203
**TN**	487	326
**FP**	39	200
**Sensitivity**	0.406844106	0.614068441
**Specificity**	0.912389381	0.62300885
**Accuracy**	0.66634981	0.616920152

For a study of an exploratory nature like this, prediction methods with low sensitivity could be counterproductive, since it would essential mean possibility of losing out lots of potential compounds during initial screening stages without any scope of being considered for its therapeutic application for oral cancer during the downstream analytical process. We therefore, selected SVM classifier for prediction of anti-cancer activity of over 84 million compounds collected from ChEMBL and STITCH database (see section Compound-Target Data Sources). We decided to leverage high specificity of CDRUG to identify possible false positives among the list of potential compounds obtained at the end of this study.

#### Prediction of Anticancer Activity

The compounds collected from ChEMBL and STITCH database (see section *Compound-Target Data Sources*) were given as input to the SVM classifier for the prediction of anticancer activity. There were over 82.84 million compounds from STITCH, and over 1.4 million compounds from ChEMBL databases. Various analytical steps involved in preprocessing (like feature extraction) and SVM prediction, have certain physical memory and CPU requirement which is determined by the size of a dataset and complexity of underlying algorithm, because of these constraints, it was not possible to analyze the whole dataset of over 84 million compounds all at once. After a couple of initial trial runs of prediction workflow with varied sized subsets of the compound dataset, we were able to find upper threshold of 2.6 million compounds which can be analyzed in the desktop with 4GB memory (with 4 cores).

The number of compounds in the dataset, obtained from STITCH (~82.84 million) were marginally above the required threshold of 2.6 million supported by system configuration used in the current study. The compound dataset from STITCH was, therefore, divided into manageable chunks, each consisting of ~2.6 million compounds, with the help of Linux ‘split’ command. The data chunks, thus obtained were given as input for anti-cancer activity prediction by SVM classifier (see section *Prediction Process* for the steps involved in prediction by SVM classifier). After all the data chunks were processed by SVM classifier, prediction results obtained from the individual chunks were consolidated as a single output file. Compound dataset collected from ChEMBL consisted of 1.4 million compounds, which were given as input for anti-cancer activity prediction by SVM classifier (see section *Prediction Process* for the steps involved in prediction by SVM classifier).

#### Association of Predicted Anticancer Compounds with Targets

Target information for the compounds derived from the STITCH database [16] were compiled by parsing the file ‘9606.protein_chemical.links.v4.0.tsv.gz’, downloaded during the data collection stage of the current study (see section *STITCH—Chemical-Protein Interaction Database*). STITCH database stores target information as an Ensembl protein identifier, we have used gene2ensembl file downloaded from NCBI to covert Ensembl ID to the corresponding Entrez Gene ID. The chemical-protein interactions in STITCH database are arranged into four categories, viz. (i) experimental: interaction information collected from experimental databases like ChEMBL [15], (ii) database: interaction information is collected from manually curated drug-traget databases like DrugBank [3], (iii) textmining: interactions gathered from literature based on co-occurrence text-mining and Natural Language Processing (NLP), and (iv) predicted: interaction information predicted as explained by Kuhn et al. [16]. In the current study, we have considered only those chemical-protein interactions, which are of categories ‘experimental’ or ‘database’, with an objective of including interactions collected from the most reliable sources. The interaction information of 449,666 compounds from STITCH database was collected, which satisfy the source constraint (experimental/database), and were, thus used internally for extracting target information of STITCH compounds which were predicted to have anticancer activity by SVM classifier.

Target information for the ChEMBL compounds predicted to have anticancer activity by the SVM classifier was retrieved by querying ChEMBL database. Bioactivity data was mined by querying ‘activities’ table, and protein assays with IC50 value of ≤ 1 μM or 1000 nM, was used as a criteria to associate target(s) with the compound. Detailed steps involved in extraction of target information of ChEMBL compounds can be found in perl script ‘chemdb_targets_pl.txt’, available as online supplementary material (S13 Text). UniProt ids were mapped into corresponding Entrez Gene ID with the help of idmapping.tab file (available for ftp download at ftp://ftp.uniprot.org/pub/databases/uniprot/current_release/knowledgebase/idmapping/by_organism/HUMAN_9606_idmapping_selected.tab.gz).

The compounds present in ChEMBL and STITCH are not mutually exclusive, therefore, there was a possibility of existence of duplicate compounds, albeit with a different ids (ChEMBL ID & STITCH compound id) in the list of predicted anti-cancer compounds. In the current analysis, duplicate compounds were identified based on similarity in their SMILES strings. The methods like calcfp() along with bitwise OR operator ‘|’, implemented in python library PyBel [27] were used to calculate the Tanimato coefficient [28]. A Tanimato coefficient of 1 was used for the identification of duplicate compounds. The targets of duplicate compounds were merged by taking the union of the targets between duplicate compounds, for an instance, compound A (targets 1,2,3), compound B (targets 2,5,6,8) and compound C (targets 3,9) were identified as identical (Tanimato coefficient of 1), such compounds were merged as a single record with a target list as 1,2,3,5,6,8,9.

### Partial Least Square Regression (PLSR) based Score Computation

Methods like multiple linear regression (MLR) are well suited for scenarios, when the factors (or variables) are few in number, are not collinear (or significantly redundant), and their relationship with responses is well-defined, however, MLR turns out to be inefficient when any of these preconditions are not met. The training data used in the current study has many variables which may be highly collinear, therefore, application of MLR to build a regression model would be inappropriate. Partial Least Squares (PLS) is an effective and robust alternative to MLR, especially, in real life scenarios when data often have many, possibly collinear, predictor variables and relatively few samples. In the current study, we have used R-package ‘pls’ for partial least square regression [29].

#### Computation of Feature Weights

The data collected from DrugBank, and literature related by natural compounds (see section *Drug-Target data*) was used to train partial least squares regression model. Data from the file ‘DB_others.txt’ (see S2 Text), ‘DB_cancer.txt’ (see S1 Text) and ‘Nat_Anticancer.txt’ (see S4 Text) was combined and transformed into the training matrix to be used for PLS regression modeling. The compounds were divided into three classes based on their ICD10 codes and were represented as response variable/target value/class: ‘1’ → compounds with activity against oral cancer, ‘0’ → compounds with activity against cancer types other than oral cancer and ‘-1’ → compounds without anti-cancer activity. Compounds like resveratrol, which are active against various cancers, including oral cancer, were represented with two instances in the training matrix with two target values ‘0’ and ‘1’; this was done to ensure that no information is lost about the activity of a compound. The training data consisted of 33 compounds with activity against oral cancer (class → ‘1’). In a previously published study, we have identified a list of potential therapeutic targets for oral cancer, based on evidences gathered from the integrative study, and ability of these therapeutic targets to affect diverse cancer hallmarks involved in carcinogenesis [30]. These targets are not well represented in existing therapeutic compounds known for oral cancer, primarily because, very less number of drugs/therapeutic compounds are currently known to be active against oral cancer. In order to address this limitation, we have included an additional instance with targets reported in the previous study [30]. The data matrix used for training the partial least squares regression model can be found in the file–‘PLS_Matrix.txt’ (see S14 Text), which is available as online supplementary material. The first two columns of ‘PLS_Matrix.txt’ corresponds to compound id, and a class label (1/0/-1), respectively, and the rest of the columns indicates molecular targets represented as Entrez Gene ID; the presence or absence of a target among target profile of a compound is represented by 1 or 0, respectively, in ‘PLS_Matrix.txt’. The mapping between compound ids to their names can be found in the file ‘Compound_index.txt’ (see S15 Text), which is available as online supplementary material.

The PLS classifier is shown as below,

$${\text{y}}_{\text{i}}=\;{\text{w}}_{\text{a}}{\text{a}}_{\text{i}}+\;{\text{w}}_{\text{b}}{\text{b}}_{\text{i}}+\;{\text{w}}_{\text{c}}{\text{c}}_{\text{i}}+\;{\text{w}}_{\text{d}}{\text{d}}_{\text{i}}+\ldots .$$ (iv)

where,

‘y’ is the training score for that compound;

where “1” represents a compound with anticancer activity against oral cancer cells, “0” represents a compound with generic anticancer activity or activity against cancers other than oral cancer, “-1” represents a compound lacking any anticancer activity.

‘w’ is the weight assigned to each target gene.

‘i’ is the index (or compound id) of the compound from P_Matrix.txt

‘a’, ‘b’, ‘c’, ‘d’, … are each gene’s compound specific feature value from P_Matrix.txt, where, “1” indicates that the compound targets gene, and “0”, is otherwise.

As implied by the equation (Eq iv), the model is trained by setting the weights for each target gene, such that, those weights when combined with individual feature values (1→Presence, 0→Absence of target gene for the particular compound), across all compounds from training matrix (‘P_Matrix.txt’) should achieve the closest correspondence of the score/class (1, 0, -1) for each training compound. The ‘leave one out’ cross-validation was used to internally train the regression model. The feature weights were extracted with the help of command “loading.weights()”. The weight obtained for a target indicates its contribution towards the activity of compound for oral cancer treatment. Feature weights were computed with the help of following R code:

**> library(pls)**
**> data <- read.table('PLS_Matrix.txt', head = T, sep = "\t")**
**> y <- as.integer(data$class)**
**> x <- subset(data,select = -c(Record_ID,class))**
**> X <- as.matrix(x)**
**> weights <- as.numeric(loading.weights(plsr(y~X,ncomp = 1,validation = ‘LOO’)))**

The weight of the target gene/protein can be found in ‘PLS_Weights.txt’ (see S16 Text) and ‘PLS_Weights_gs.txt’ (targets represented as Gene Symbol, see S17 Text), available as online supplementary material.

#### Computation of Oral Cancer specific score (OC_Score)

Compounds sharing indication area, invariably have a similar target profile, because at the mechanistic level, they are targeting biological processes involved in the genesis / progression of the same disease. In the current study, we have computed a target based statistic (or score) which could help in screening compounds with activity against oral cancer. The oral cancer specific feature weights (‘PLS_Weights.txt’ see S16 Text) were used to compute score, which we named as ‘OC_Score’.

$$\text{OC}\_\text{Score }=\;\sum_{i=1}^{n}w[i]$$ (V)

where,

‘n’ is the number of targets for a query compound.

‘w[i]’ feature weight for i^th^ target.

The steps involved in computation of OC_Score, for a query compound from the training dataset is as follows:

1. Collect target profile of a query compound.
2. Extract weights for targets of a query compound from ‘PLS_Weights.txt’.
3. Compute OC_Score for the query compound by adding up the weights extracted in step 2.

*Scores thus computed for the training compounds can be found in the file ‘Score_distribution.txt’ (see S18 Text) available as online supplementary material.

Computation of OC_Score with an example is illustrated in Table 2. OC_Score is generally higher and positive when the potential of a compound to treat oral cancer is greater, and vice-versa.

**Table 2:** Computation of OC_Score.

Lycopene			Zhankuic acid C		
Gene Symbol	GeneID	Weights	Gene Symbol	GeneID	Weights
ABCA1	19	-0.001570518	BCL2	7422	0.248744521
NR1H3	10062	0.003148697	CASP3	1956	0.456548318
PPARG	5468	-0.020447379	PARP1	7010	0.177892451
**OC_Score =**		**-0.0188692**	**OC_Score =**		**0.883185289**

### Selection of Potential Compounds for Oral Cancer treatment

**Fig 5:**
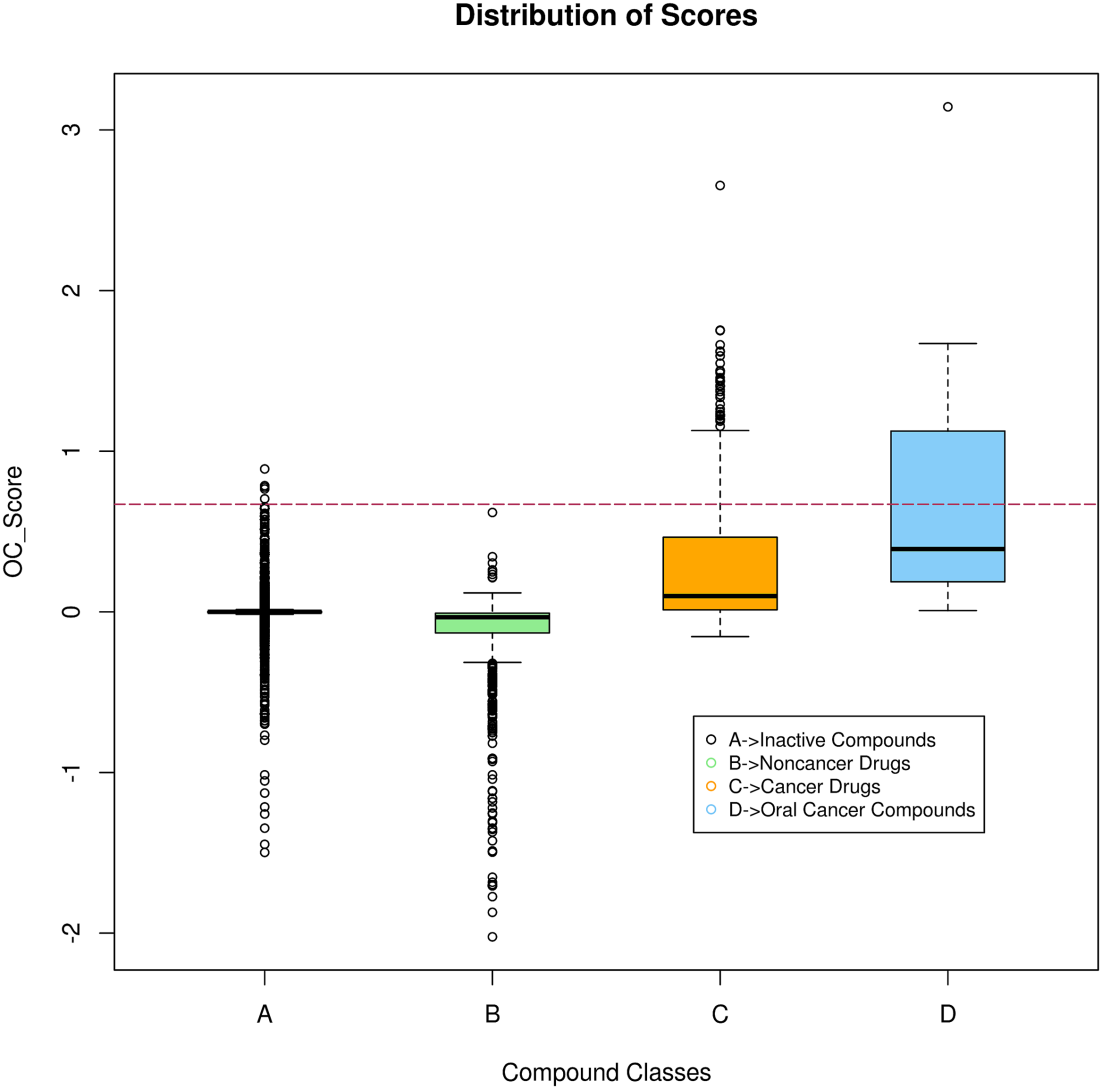
Distribution of oral cancer specific statistic ‘OC_Score’. Box-plots depicting score distribution of compounds belonging to groups formed on the basis of differences in drug indication area. Horizontal line indicates the cutoff used in the current study to select potential compounds.

OC_Scores were computed for compounds used for training the partial least squares regression matrix, these compounds were categorized into three classes, oral cancer (represented with a class label ‘1’), cancer (represented by class label ‘0’) and others (represented by the class label ‘-1’) (see S18 Text). In order to estimate background scores, we first extracted target profile of compounds from ChEMBL, which were predicted to be in-actives by SVM classifier. OC_Scores of these compounds were computed, and were considered as background scores. The scores thus obtained provided us with distribution of OC_Scores among different compound classes (Table 3). The cutoff score of ≥ 0.67 (mean score of compounds used to treat oral cancer, refer Table 3) was used for the identification of potential compounds for oral cancer treatment. It can be seen that with a cutoff value of ≥ 0.67, there is almost negligible overlap between scores of the compounds for the oral cancer treatment and the scores of the compounds used in other therapeutic areas (non-cancerous diseases) (Fig 5); barring a couple of outliers from inactive compounds, this cutoff can be regarded as efficient in filtering out non-relevant compounds.

**Table 3:** Descriptive Statistics of OC_Scores for different compound classes.

	Min.	1st Quartile	Median	Mean	3rd Quartile	Max.
**Oral Cancer**	0.00787	0.1944	0.3912	0.6699	1.12	3.144
**Cancer**	-0.8494	0.01261	0.09847	0.3059	0.4644	2.654
**Others**	-2.023	-0.1313	-0.03301	-0.1245	-0.007859	0.6187
**Inactives**	-1.497	-0.004719	0	0.00417	0.003149	0.8895

OC_Score was computed for the non-redundant list of compounds predicted to have anti-cancer activity by an SVM classifier (see section *Association of Predicted Anticancer Compounds with Targets* for details about the list of active anti-cancer compounds). The cutoff score of ≥ 0.67 was used to select compounds from this list, which were regarded as potential compounds for oral cancer treatment.

#### Annotation of List of Potential Compounds

Additional information like the associated GI50 value, physicochemical properties, etc. was gathered for the list of potential compounds. GI50 is the concentration of test compound required to cause a 50% reduction in the proliferation of cancer cells. PubChem bioactivity database assigns bioactivity outcome (as ‘active’) using a ≤ 50μM cutoff based on readouts such as IC50, GI50, EC50 etc. [31]. In the current study, we have mined GI50 values for the list of potential compounds for oral cancer treatment (from ChEMBL/PubChem bioassay db), and used the same cutoff of ≤ 50μM to associate active GI50 assays against them. Compounds from STITCH database are derived from PubChem, therefore, their compound ids can be converted into corresponding PubChem Compound ID (or cid) [16]. Therapeutic compounds collected from DrugBank and natural sources (see section *Drug-Target Data*) were associated with PubChem cid. Bioassay data from PubChem BioAssay, can be collected with the help PUG/REST structured URLs in the following format: http://pubchem.ncbi.nlm.nih.gov/rest/pug/compound/cid/<ListOfCIDs>/assaysummary/CSV

BioAssay data for compounds from ChEMBL were collected by querying ChEMBL database. For an instance, GI50 values of active bioassays associated with resveratrol (chembl_id:CHEMBL165), were obtained with the help of following SQL query: “*select a*.*assay_id*, *b*.*standard_type*, *b*.*standard_value*, *b*.*standard_units*, *a*.*description from assays a*, *activities b where a*.*assay_id = b*.*assay_id AND b*.*standard_type = 'GI50' AND ((b*.*standard_value< = 50 AND b*.*standard_units LIKE 'u%') OR (b*.*standard_value< = 50000 AND b*.*standard_units LIKE 'n%')) AND b*.*molregno = (select molregno from molecule_dictionary where chembl_id = ‘CHEMBL165');*”

Various physicochemical parameters for the list of potential compounds were computed with the help of python library ‘PyBel’ [27]. The parameters, thus computed, were used for estimation of Lipinski’s ‘rule of 5’ which predicts that poor absorption or permeation is more likely when there are more than 5 hydrogen-bond donors, 10 hydrogen-bond acceptors, the molecular weight is greater than 500 and the Log P is greater than 5 [32]. Topological polar surface area (TPSA) was another physicochemical property which was calculated in the current study. TPSA is considered to be a good predictor of oral bioavailability. The molecule with TPSA of ≥ 140 Å^2^ are likely to exhibit poor intestinal absorption [33]. ChEMBL tags compound with value ‘Y/N’ (for the field “MedChem Friendly”) based on the absence / presence of functional groups which are not desirable from the medicinal chemistry perspective (refer link https://www.ebi.ac.uk/chemblntd/glossary). We have collected SMARTS string of the undesirable functional groups used by ChEMBL, and used them to screen the list of potential compounds. This screening was conducted with the help of python script ‘Check_MedChemFriendly_py.txt’ (see S19 Text) available online as supplementary material. The smiles strings of the list of potential compounds obtained in the current study were given as a input to CDRUG [17], with an objective to flag out possible false positives.

The common names of the compound derived from PubChem/ChEMBL were retrieved from respective databases. PubChem allows retrieval of compound names for an input list of compound ids (PubChem CID), with the help of PUG/REST structured URLs in following format: https://pubchem.ncbi.nlm.nih.gov/rest/pug/compound/cid/<ListOfCIDs>/description/XML. The XML file consists of compound name enclosed between <Title>*cname*</Title> tags, the perl library ‘XML::Simple’ was used to parse xml file and collect common names of the list of compounds with PubChem cid. The common names of the compounds from ChEMBL database can be obtained by querying ChEMBL database table ‘molecule_dictionary’ and is available in the field ‘pref_name’. For an instance, common name for compound id ‘CHEMBL165’ (compound id for resveratrol) can be retrieved with the help of SQL query ‘*select pref_name from molecule_dictionary where chembl_id = “CHEMBL165”*’.

The NCBI-PubMed was queried to get overview of supporting publication evidences for potential compounds, identified in the current study. The PubMed search was conducted with the help of specific queries, structured to retrieve relevant articles which mention the therapeutic role of a compound with respect to oral cancer. For an instance, following PubMed search query can be used to retrieve research articles relevant to the therapeutic role of resveratrol with respect to oral cancer: “(Resveratrol [TIAB] AND mouth neoplasms[MH] AND (Therapeutic [TIAB] OR Therapy [TIAB] OR Treatment[TIAB])) NOT Review[PT]”.

## Results and Discussion

The SVM classifier predicted 34,597,939 (~34.60 million) compounds as anti-cancer compounds among 82.84 million compounds collected from STITCH database. Among these predicted anti-cancer compounds (~34.60 million), the target information of 231,123 compounds meeting internal selection criteria was collected (interaction gathered from pathways/databases were considered, see the section *Association of Predicted Anticancer Compounds with Targets* for details). The SVM classifier predicted 620,273 compounds to be anti-cancer compounds among the 1.4 million compounds from ChEMBL database. Among these predicted anti-cancer compounds (620,273 compounds), the target information of 91,795 compounds meeting internal selection criteria was collected by querying ChEMBL database (protein assays with IC50 value of ≤ 1 μM, see the section *Association of Predicted Anticancer Compounds with Targets* for details). The unique list of compounds with target profile was obtained by merging compound-target dataset of predicted anticancer compounds from ChEMBL (91,795 compounds) and STRING (231,123 compounds) database (Tanimato coefficient of 1 between compounds from two data sources was used to identify duplicate compounds, see the section *Association of Predicted Anticancer Compounds with Targets* for details). The merged list of predicted anti-cancer compounds consisted of 199,572 compounds.

**Fig 6:**
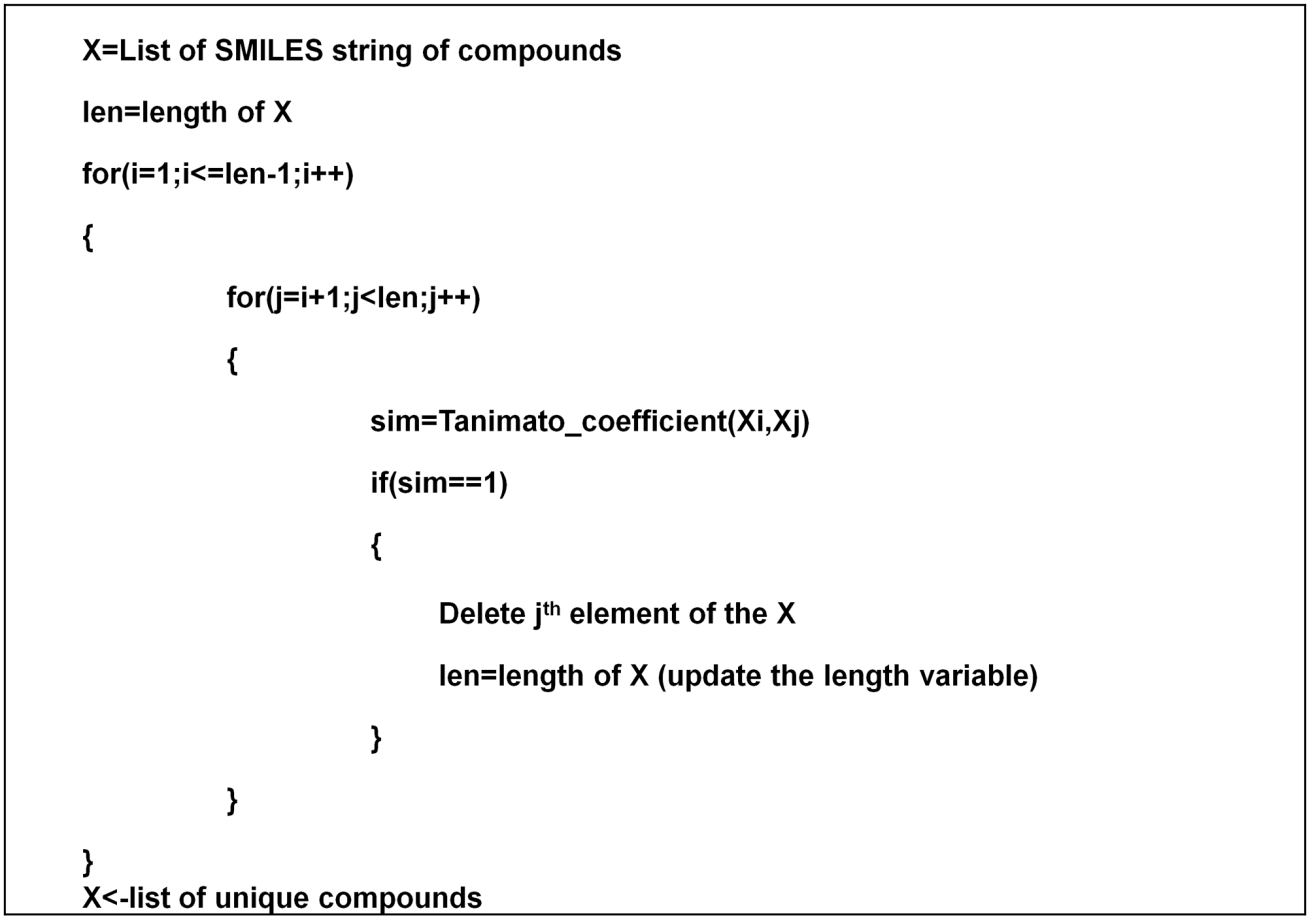
Algorithm for finding duplicates in the list of compounds.

**Fig 7:**
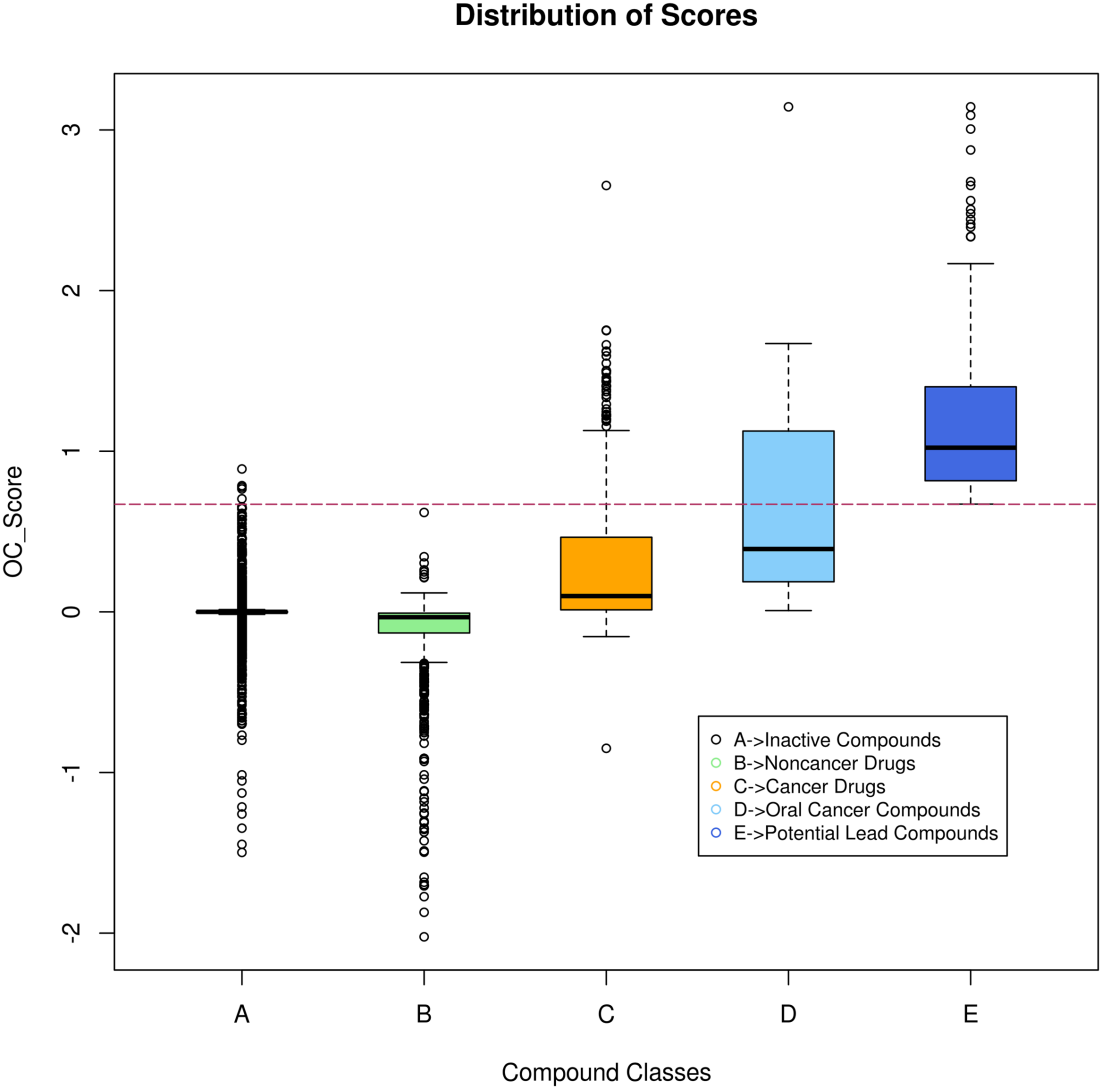
Distribution of oral cancer specific statistic ‘OC_Score’. Box-plots depicting score distribution of compounds belonging to different groups, compared with those identified as potential compounds for oral cancer treatment. Horizontal line indicates the cutoff used in the current study to select potential compounds.

The target weights obtained from the partial least squares regression modelling was used to compute oral cancer specific statistic ‘OC_Score’, for the merged list of predicted anti-cancer compounds. The OC_Scores thus obtained for the merged list of predicted anti-cancer compounds can be found by uncompressing zipped file ‘Ch_Pub_consolidate_list.zip’ (see S20 Text) available online as supplementary material. A total of 311 compounds were selected, based on OC_Score cutoff of ≥ 0.67, and were regarded as compounds with potential to treat oral cancer. Quality check of this list, of 311 compounds was conducted and it was observed that quite a few duplicate compounds from STITCH database were still present in this list. Few compounds in STITCH database are represented by two records which correspond to its stero-specific and flat structure (refer to: http://stitch.embl.de/download/README). Such duplicate compounds were identified and removed based on their structural similarity to retrieve the list of unique compounds (Fig 6). After removal of duplicates among this list, we got 218 compounds (80 compounds from ChEMBL, and 138 compounds from PubChem/STITCH database). The compounds used for building partial least squares regression model were also interrogated to check their potential to treat oral cancer (see S18 Text). A total of 100 compounds with an OC_Score cutoff of ≥ 0.67 were collected, these consisted of compounds from DrugBank database [3] and manually curated list of plant based natural products with anticancer activity. The list of potential compounds derived from different sources (ChEMBL, STITCH / PubChem compounds, DrugBank and manually curated anticancer natural products) were consolidated, and compound duplicates, if any, were removed from this list. The consolidated list has 288 potential compounds with OC_Scores ranging from 0.67 to 3.14 (Fig 7). These compounds were annotated with associated attributes related with their bioactivity against cancer cells and physicochemical parameters (see section *Annotation of List of Potential Compounds*). The detailed information about active BioAssays associated with these compounds can be found in ‘GI50_BioAssays.xlsx’ (see S21 Text), available as online supplementary material. The possible false positives were identified by CDRUG tool [17]; the result of this analysis can be found in ‘Lead_cmpds_cdrug_result.txt’ (see S22 Text), available as online supplementary material.

The complete list of these compounds along with associated annotations can be found in ‘OC_LeadCompounds_1.1.xlsx’ (see S23 Text), available as online supplementary material. The statistic regarding potential compounds identified in the current study can be found in Table 4.

**Table 4:** Details about potential compounds for oral cancer treatment identified in current study.

Description	Counts#
Total no. of Compounds	288
Active BioAssays (GI50)	53
Natural Products	155
Compounds with Oral Cancer Evidences	85
Medicinal Chemistry Friendly	229
CDRUG (Significant)	116
Rule of Five	188

It can be seen that, majority of compounds obtained are of natural origin. The annotations associated with the compounds in this spreadsheet (see S23 Text) were utilized, to further filter the list of potential compounds. Within this list, the potential compounds were selected based on following criteria:

1. BioAssay Data: Compound with significant bioactivity against cancer cells has a higher probability to successful transition into anti-cancer drug.
2. Significance value (p value) cutoff of ≤ 0.05.
3. Optimal physicochemical properties or compound satisfying rule of five criteria [32], and without undesirable functional groups.
4. Target profile relevant to oral cancer, measured by OC_Score. The compounds with higher OC_Scores generally have higher potential to treat oral cancer.
5. Prior evidence of compound’s activity against oral cancer cells.

**Fig 8:**
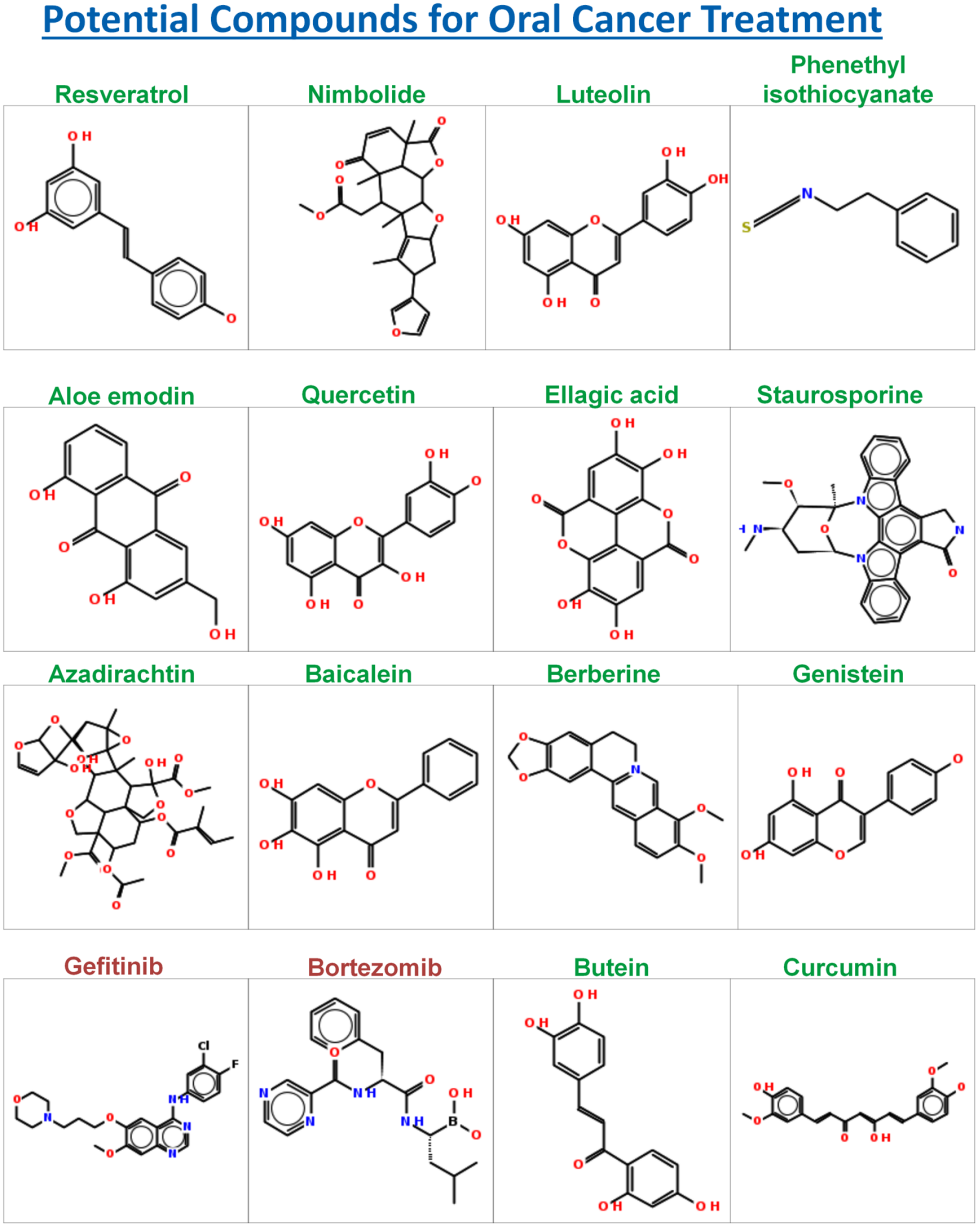
Compound structures of potential compounds with supporting evidences about their activity against oral cancer. The name of compound with natural origin is distinctly highlighted with green color.

**Fig 9:**
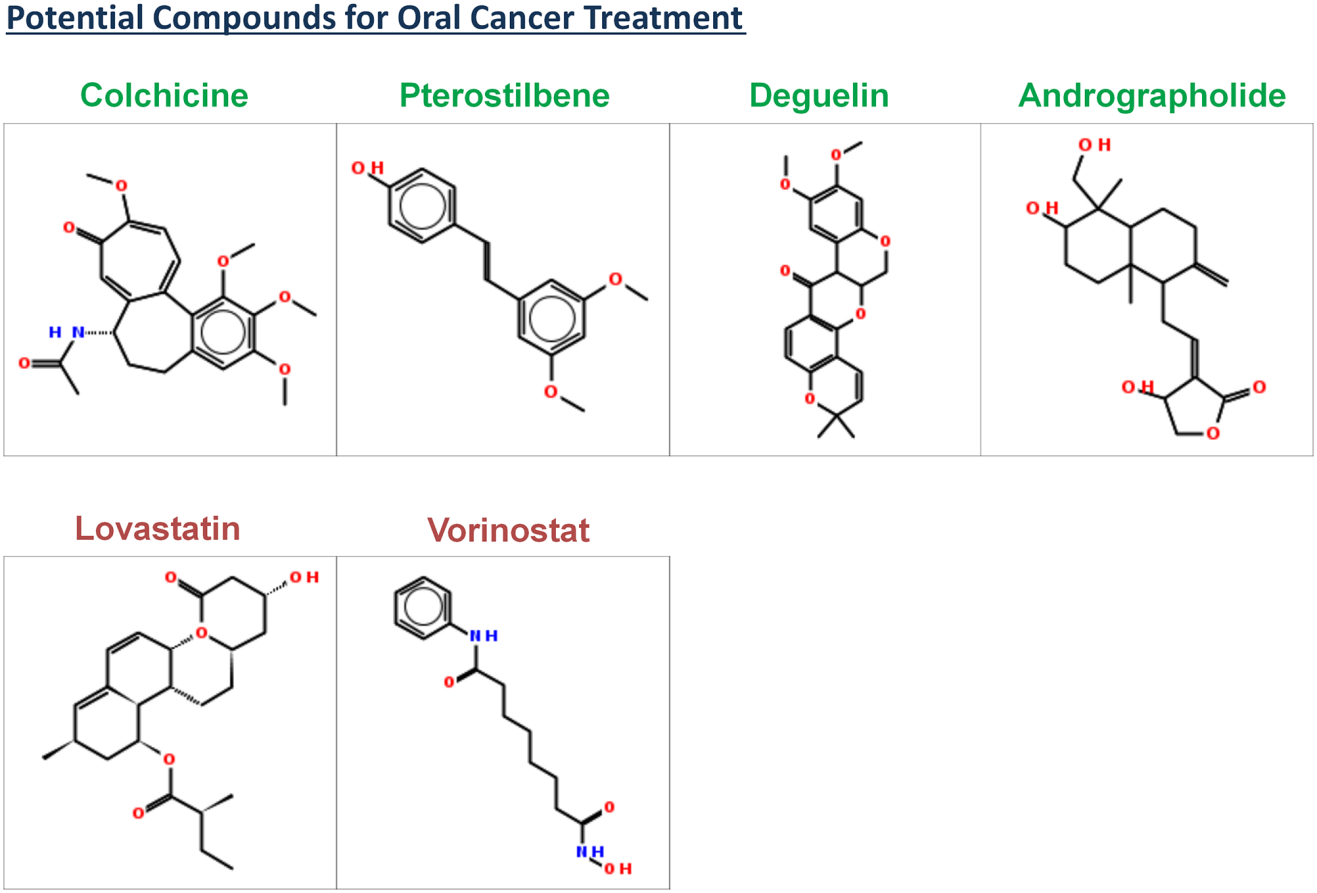
Compound structures of potential compounds with supporting evidences about their activity against oral cancer. The name of compound with natural origin is distinctly highlighted with green color.

**Fig 10:**
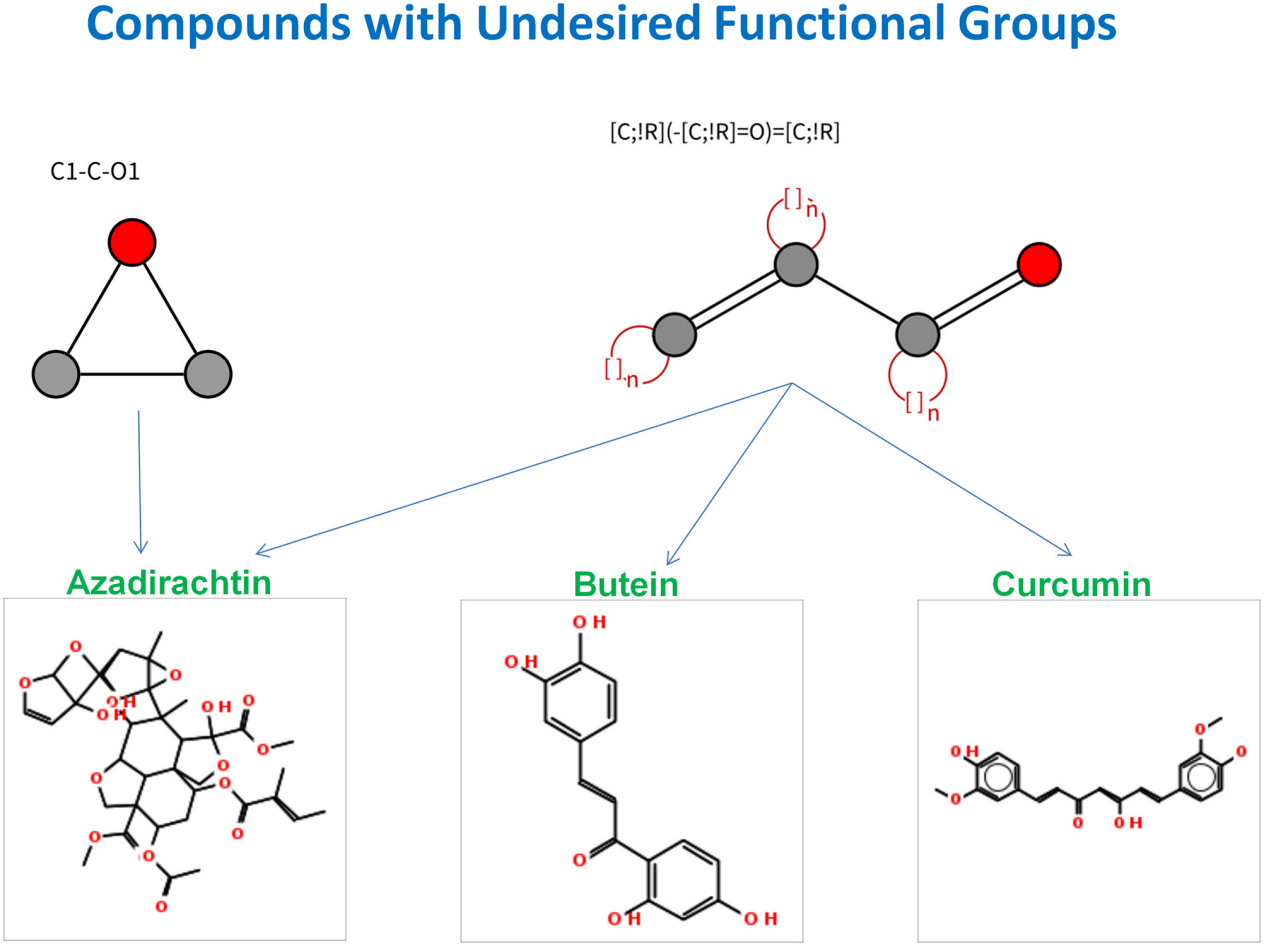
Compound with undesirable functional groups. From the medicinal chemistry perspective, these functional groups in compounds are undesirable.

The manual review of the set of articles retrieved by specific PubMed queries (see section *Annotation of List of Potential Compounds*), used for annotating the list of compounds was conducted to validate the therapeutic role of compounds with respect to oral cancer. Some of the potential compounds satisfying the aforementioned majority of the selection criteria are resveratrol, nimbolide, luteolin, phenethyl isothiocyanate, aloe emodin, quercetin, ellagic acid, staurosporine, bortezomib, gefitinib, genistein, biocalien, berberine, colchicine, lovastatin, vorinostat, pterostilbene, deguelin, and andrographolide (Figs 8 and 9). Compounds like butein, curcumin, paclitaxel, docetaxel and azadirachtin represent the set of compounds with a potential to treat oral cancer albeit with shortcomings in physicochemical parameters. Compounds like butein, curcumin, and azadirachtin, were identified to be not medicinal chemistry friendly because of the presence of undesired functional groups. SMARTS strings of undesired functional groups present in these compounds were illustrated with the help of visualization tool—SMARTSviewer [34] (Fig 10).

Resveratrol is a natural product identified in the current analysis with a high potential to treat oral cancer. It has shown activity against various cancers, including oral cancer [35]. The diversity of molecular targets for resveratrol suggests that it controls cancer through multiple pathways. Its ‘OC_Score’ is second highest among all the compounds identified in the current analysis. Its physicochemical properties are well within the range reported for the therapeutically active drugs [32], thus making it an ideal candidate for small molecule based targeted therapy for oral cancer. Nimbolide is a natural bioactive compound (extracted from neem tree) identified in the current analysis with a high potential to treat oral cancer. It has been reported to induce apoptosis and inhibit cell proliferation in an animal model for oral carcinogenesis [36]. It controls growth of cancer cells by modulating range of targets, including p53, Survivin, and Caspases involved in key hallmark events such as cell proliferation and apoptosis. Luteolin is another natural compound identified in the current study with a potential to treat oral cancer. It has been reported to induce apoptosis in cancer cells [37]. It has shown activity against various cancers (see S21 Text). Phenethyl isothiocyanate a natural compound identified in the current analysis as potential compound has been reported to suppress invasion of oral squamous carcinoma cell [38]. Aloe emodin a natural compound identified in the current analysis has been reported to regulate growth of oral cancer cells by disrupting DNA repair mechanism [39]. Quercetin is a natural compound identified in the current analysis with a potential to treat oral cancer. It has significant bioactivity against various cancer cell-lines (see S21 Text), including oral cancer [40]. It targets proteins involved in diverse hallmark events like metastasis, cell proliferation and apoptosis. Ellagic acid is another natural bioactive compound identified with the potential to treat oral cancer. It has significant bioactivity against various cancer cell-lines (see S21 Text), including oral cancer [41], [42]. Staurosporine is a natural compound identified in the current study with a potential to treat oral cancer. It is active against various cancer cell lines (see S21 Text), including oral cancer [43]. Baicalein is a natural compound identified in the current analysis with a potential to treat oral cancer. It has significant bioactivity against various cancer cell-lines (see S21 Text), including oral cancer [44]. Its molecular targets are involved in key hallmark events like apoptosis, and cell-growth. Berberine is a natural compound identified in the current analysis with a potential to treat oral cancer. It has significant bioactivity against various cancer cell-lines (see S21 Text), including oral cancer [45]. Genistein is a natural compound identified in the current analysis with a potential to treat oral cancer. It has significant bioactivity against various cancer cell-lines (see S21 Text), including oral cancer [46]. Gefitinib was identified as a potential compound in the current study; it is active against various cell-lines, (see S21 Text) including oral cancer [11]. Bortezomib was identified as a potential compound in the current study; it is active against various cell-lines (see S21 Text) including oral cancer [47]. Lovastatin is a hypolipidemic agent, which was identified as potential compound in the current study. It has significant bioactivity against various cancer cell-lines (see S21 Text), including oral cancer [48]. Vorinostat is a histone deacetylase inhibitor, which is also known as suberanilohydroxamic acid (abbreviated as SAHA); it was identified as potential compound in the current study. It has significant bioactivity against various cancer cell-lines (see S21 Text), including oral cancer [49, 50]. Pterostilbene was identified as a potential compound in the current study, it has significant bioactivity against various cancer cell-lines. It was reported to suppress invasion of oral cancer cells by inhibiting the expression of MMP-2 [51]. Deguelin was identified as a potential compound in the current study, It was reported to T suppress the invasion and migration of oral cancer by downregulating TNF-alpha-induced NF-kB signaling [52]. Andrographolide was identified as potential compound in the current study. It has significant bioactivity against various cancer cell-lines (see S21 Text). It was reported to inhibit oral squamous cell carcinogenesis through NF-kB inactivation [53]. Colchicine was identified as potential compound in the current study. It has significant bioactivity against various cancer cell-lines (see S21 Text), including oral cancer [54]. In a comparative study it was found to have better therapeutic potential when administered to patients with Oral Submucous Fibrosis (OSF), which is pre-cancerous condition of oral mucosa [54].

Curcumin was identified as a potential compound in the current study; it is active against various cell-lines (see S21 Text) including oral cancer [55]. Azadirachtin is another neem based bioactive compound identified in the current study as a potential compound for oral cancer treatment. It has been reported to induce apoptosis and inhibit cell proliferation in animal model for oral carcinogenesis [36], however the presence of undesirable functional groups (Fig 9), and its poor physicochemical properties may limit its chance of clinical success.

To our knowledge, this is the first study in which such a huge number of compounds (~84 million) were interrogated in an unbiased manner for the possibility of their application in oral cancer treatment. The underlying datasets used for prediction were carefully selected to make reliable and biologically meaningful predictions. We have carefully reviewed list of 288 potential compounds obtained in the current study, most of them are actively investigated for treatment of various cancers. The result presented herein, reaffirms the indispensable role played by natural products in cancer therapeutics. The poor bioavailability of natural products is often a common challenge in their clinical application [56]; it can be addressed through various structural optimization, or through the use of novel and innovative drug delivery systems [57].

The analytical approach presented here is robust and powerful, which can be applied to generate potential leads for other therapeutic areas, as well. The SVM classifier trained on functional groups based features has proven to be an effective filter to mine anticancer compounds from a huge pile of compounds. The data used for training the partial least squares regression model is carefully mined from reliable sources, and can be used by researchers working in the field of other cancer types by appropriately changing the class label. The analytical framework presented for the regression based computation of target weights is flexible enough to include prior knowledge about targets known to be associated with the disease. The computation of statistic specific to disease type is logical and straightforward, and has proven to be powerful in generating potential leads. The score is computed in such a way that the target relevant to oral cancer, contributes towards the increment of the compound’s score, whereas, the irrelevant target or off-target brings down the overall score; thus the compounds with high scores should be more effective with minimal toxicities (which are mostly attributed to the compound’s off-targets).

The current study has identified potential compounds for oral cancer treatment. Some of the potential therapeutic compounds identified in the current study are resveratrol, nimbolide, lovastatin, bortezomib, vorinostat, berberine, pterostilbene, deguelin, andrographolide, and colchicine. The compounds identified in this study are based on relative importance of their targeted protein(s) in oral carcinogenesis, thus making them strong candidate for oral cancer treatment. Our future efforts would be to understand the mechanism of action of compounds identified in this study by linking their target profile with molecular pathways, cancer hallmark events.

## Supporting Information

S1 Text List of anticancer drugs extracted from DrugBank database. File contains following columns: (i) "DrugName"→Common name of the drug; (ii) "ICD10"→ICD10 disease code(s) of indication(s) for the drug; (iii) "CAS_No"→CAS Registry no. of chemical compound, and be searched @ www.commonchemistry.org, this field is left blank for biotech drugs; (iv) "PubChemCID"→NCBI-PubChem CID, can be searched @ https://pubchem.ncbi.nlm.nih.gov/, this field is left blank for biotech drugs; (v) "Smiles"→SMILES string of chemical compound; (vi) "Targets "→Entrez GeneID of the drug target(s); (vii) "Targets(GeneSymbol)"→Gene Symbol of the drug target(s).
(TXT)

S2 Text List of Drugs from other indication areas (i.e. non anti-cancer drugs) extracted from DrugBank database. File contains following columns: (i) "DrugName"→Common name of the drug; (ii) "ICD10"→ICD10 disease code(s) of indication(s) for the drug; (iii) "CAS_No"→CAS Registry no. of chemical compound, and be searched @ www.commonchemistry.org, this field is left blank for biotech drugs; (iv) "PubChemCID"→NCBI-PubChem CID, can be searched @ https://pubchem.ncbi.nlm.nih.gov/, this field is left blank for biotech drugs; (v) "Smiles"→SMILES string of chemical compound; (vi) "Targets "→Entrez GeneID of the drug target(s); (vii) "Targets(GeneSymbol)"→Gene Symbol of the drug target(s).
(TXT)

S3 Text List of Natural Anticancer compounds for various cancer-types. File contains following columns: (i) "ICD10"→ICD10 disease code(s) of the cancer-type, corresponding name of cancer-type can be searched @ http://apps.who.int/classifications/icd10/browse/2010/en; (ii) "References"→External reference id (PubMed ID/DOI) of the research article used for inferring relationship between cancer and natural compound; (iii) "Compound_Name "→Name of the natural anti-cancer compound; (iv) “PubChem_cid”→NCBI-PubChem CID; (v) "CAS_No."→CAS Registry no. of chemical compound; (vi) "Target(s)"→ Entrez GeneID of the compound’s molecular target(s); (vii) ‘Target_Ref’→External reference id (PubMed ID/DOI) of the research article used for inferring relationship between natural compound and its molecular target(s).
(TXT)

S4 Text List of Natural Anticancer compounds manually extracted from published articles. File contains following columns: (i) "DrugName"→Common name of the natural compound; (ii) "ICD10"→ICD10 disease code(s) of indication(s) for the natural compound; (iii) "CAS_No"→CAS Registry no. of the compound; (iv) "PubChemCID"→NCBI-PubChem CID; (v) "Smiles"→SMILES string of the compound; (vi) "Targets "→Entrez GeneID(s) of the compound’s target(s); (vii) "Targets(GeneSymbol)"→Gene Symbol(s) of the compound’s target(s).
(TXT)

S5 Text SMARTS pattern of the functional groups used in the current study. The file contains following columns: (i) ‘FuntionalGroup’→Functional group name; (ii) ‘SMARTS’→SMARTS representation of the functional group.
(TXT)

S6 Text SVM Model Building files. Compressed file, consisting of intermediate files generated during the grid search process, and final model/classifier ‘cancer.model’ used in the current study.
(ZIP)

S7 Text List of anti-cancer compounds used for method validation/comparison. The file consists of two columns, the first column is a SMILES string of the compound, and the second column is a compound name.
(TXT)

S8 Text List of non anti-cancer compounds used for method validation/comparison. The file consists of two columns, the first column is a SMILES string of the compound, and the second column is a compound name.
(TXT)

S9 Text Anticancer activity prediction on validation dataset by CDRUG. In the current study, compounds with p_value of ≤ 0.05 were considered to have anti-cancer activity.
(TXT)

S10 Text Feature Matrix of the compounds from validation dataset. First column of this file represents a compound name, rest of the columns except last represents functional groups, and the last column (‘Active’) can have following value: 1→ indicates compound has anti-cancer activity, and -1→ indicates compound doesn’t have anti-cancer activity; Each row of this file represents feature vector of the compound, it consist of list of binary values (0→indicates absence of functional group in the compound; 1→ indicates presence of functional group in the compound).
(TXT)

S11 Text Feature matrix of validation dataset transformed into the format used by SVM classifier for prediction. (TXT)

S12 Text Anti-cancer activity prediction on validation dataset by SVM classifier. ‘1’→ indicates compound predicted to have the anti-cancer activity, ‘-1’→ indicates compound predicted to lack the anti-cancer activity.
(TXT)

S13 Text Perl script used for extracting targets for the list of compound from ChEMBL database. Script mines target information by querying locally installed ChEMBL database.
(TXT)

S14 Text Input matrix used for the partial least squares regression. First column of this file represents the compound id, the second column represents a compound’s class (which can assume three values: 1→ indicates compound has activity against oral cancer, 0→ indicates compound has activity against cancer-types other than oral cancer, and -1→ indicates compound doesn’t have anti-cancer activity), and the rest of the columns represents the presence (indicated by 1) or absence (indicated by 0) of target associated with the compound.
(TXT)

S15 Text Details of compounds used for the partial least squares regression. File contains following columns: (i) ‘Record_ID’→ID of the compound used for partial least squares regression, this id can be used for cross-referencing the record in PLS_Matrix.txt (or S14 Text); (ii) ‘class’→: 1→ indicates compound has activity against oral cancer, 0→ indicates compound has activity against cancer-types other than oral cancer, and -1→ indicates compound doesn’t have anti-cancer activity; (iii) ‘CompoundName’→common name of the compound; (iv) ‘Targets’→Entrez GeneID of the compound’s target(s).
(TXT)

S16 Text Target weights obtained from the partial least squares regression. File contains following columns: (i) ‘features’→Entrez Gene ID of the target; (ii) ‘weights’→weights obtained from pls modeling.
(TXT)

S17 Text Target (with Gene Symbol) weights obtained from the partial least squares regression. The file contains following columns: (i) ‘features’→Gene Symbol of the target; (ii) ‘weights’→weights obtained from pls modeling.
(TXT)

S18 Text Computation of OC_Score for the list of compounds used for partial least squares regression. File contains following columns: (i) ‘Class’→ 1→ indicates compound has activity against oral cancer, 0→ indicates compound has activity against cancer-types other than oral cancer, and -1→ indicates compound doesn’t have anti-cancer activity; (ii) ‘CompoundName’→common name of the compound; (iii) ‘OC_Score’→oral cancer specific statistic/score.
(TXT)

S19 Text Python script used for checking whether a compound is a medicinal chemistry friendly or not?. Script to check if compound is medical chemistry friendly based on absence or presence of undesirable functional group(s).
(TXT)

S20 Text Computation of OC_Score for the list of compounds predicted by the SVM classifier to have the anti-cancer activity. The file obtained after uncompressing this file contains following columns: (i) ‘CompoundId’→ChEMBL ID or STITCH compound id; (ii) ‘Smiles’→SMILE string of the compound; (iii) ‘Targets’→Entrez GeneID(s) of the compound’s target(s); and (iv) ‘OC_Score’→oral cancer specific statistic/score.
(ZIP)

S21 Text Cancer specific bioactivity records of the potential compounds. The file contains following columns: (i) ‘CompoundName’→common name of the compound; (ii) ‘CompoundId’→ChEMBL ID or PubChem compound id; (iii) ‘CompoundSource’→Source of the compound from which it was derived in the current study, it can have one of the three sources i.e ChEMBL or PubChem or from DrugBank/Natural compound list collected in the study; (iv) ‘BioAssaySource’→Reference bioassay database (ChEMBL or PubChem BioAssay); (v) ‘AssayID’ →Record ID of the assay, it can be used to search the record in corresponding bioassay database; (vi) ‘AssayType’→GI50 assay; (vii) ‘AssayValue’→activity value for the bioassay; (viii) ‘AssayUnits’→unit used for activity measurement; (ix) ‘AssayDescription’→ Description of the assay.
(XLSX)

S22 Text Anticancer activity prediction on the list of potential compounds by CDRUG. In the current study, compounds with p_value of ≤ 0.05 were considered to have anti-cancer activity.
(TXT)

S23 Text List of potential therapeutic compounds for oral cancer. Contains four sheets 'Potential_Compounds', ‘ChEMBL_Compounds’, ‘PubChem (STITCH db)_Compounds’, and ' NAT_DB'. ''Potential_Compounds' contains list of therapeutic compounds for oral cancer found to be most potential. This sheet contains following columns: (a) ‘CompoundName’→common name of the compound; (b) ‘CompoundId’→ChEMBL ID or PubChem compound id; (c) ‘CompoundSource’→Source of the compound from which it was derived in the current study, it can have one of the three sources i.e ChEMBL or PubChem or from DrugBank/Natural compound list collected in the study; (d) ‘Smiles’→SMILE string of the compound; (e) ‘Targets’→Gene Symbol(s) of the compound’s target(s); (f) ‘OC_Score’→oral cancer specific statistic/score; (g) ‘Active_GI50_assay’→ Compound’s bioactivity against cancer cells, this field can have two values:’1’→ indicates compound was report to have active GI50 assay, or ‘0’→ indicates compound was not reported to be active in GI50 assay; (h) ‘NaturalCompound’→Compound with natural origin are marked as ‘1’, and ‘0’ indicates otherwise; (i) ‘OC_train_compound’→ Compound known to be active against oral cancer and which were used during regression process are marked as ‘1’, and ‘0’ indicates otherwise; (j) ‘Med_chem_friendly’→ Compound which doesn’t have any undesirable functional groups are marked as ‘Y’ indicating that it is medicinal chemistry friendly, and ‘N’ indicates otherwise; (k) ‘p_value’→p value for the compound computed by CDRUG; (l) ‘PubMed Hits #’→No. of articles related with the therapeutic role in oral cancer for the compound retrieved on querying the PubMed database; (m) ‘TPSA to NROT’→Various physicochemical properties computed for the compound, where TPSA→Topological Surface Area, HBD→Hydrogen Bond Donor, MW→Molecular Weight, logP→Partition coefficient, HBA→Hydrogen Bond Acceptor, NROT→Number of rotatable bonds; (n) ‘RuleOfFive_Pass’→ Compounds with physicochemical properties within cutoff suggested by Lipinski et al. are marked as ‘TRUE’, and ‘FALSE’ indicates otherwise. The rest of the sheets corresponds to the list of potential compounds grouped based on the source, for e.g. compounds listed in the ‘ChEMBL_Compounds’ were derived from ChEMBL database.
(XLSX)
